# Analysis on effect of psychological nursing combined with extended care for improving negative emotions and self-care ability in patients with colorectal cancer and enterostomy: A retrospective study

**DOI:** 10.1097/MD.0000000000038165

**Published:** 2024-05-24

**Authors:** Fang Liu, Kun Yao, Xiaoxiang Liu

**Affiliations:** a Hernia and Colorectal Surgery, The Second Affiliated Hospital of Dalian Medical University, Dalian, Liaoning, China.

**Keywords:** colorectal cancer enterostomy, extended care, negative emotions, psychological nursing, self-care ability

## Abstract

This study investigates the effectiveness of combining psychological nursing with extended nursing in patients with colorectal cancer who have undergone enterostomy. Conducted from January 2021 to January 2022, this retrospective study involved 78 patients split into 2 groups of 39 each. The control group received standard nursing care, while the observation group benefitted from both psychological and extended nursing. The evaluation focused on anxiety, depression, sleep quality, mental resilience, and self-care abilities. Results, 3 months postdischarge, indicated that the observation group had significantly lower scores in the Hamilton Depression Rating Scale and the Pittsburgh Sleep Quality Index, and higher scores in the Connor-Davidson Resilience Scale and the Enterostomal Self-Care Ability Scale, compared to the control group (*P* < .05). The findings suggest that integrating psychological nursing with extended care significantly improves mood, sleep quality, psychological resilience, and self-care capabilities in these patients.

## 1. Introduction

Colorectal cancer, encompassing both colon and rectal cancer, is a prevalent malignancy affecting the digestive tract. The incidence and mortality rates of colorectal cancer are on the rise, attributed to unhealthy dietary habits and lifestyles.^[1]^ According to statistics.^[2]^

In 2018, the global incidence of colorectal cancer reached 1096,600 new cases, with 880,000 fatalities Within China during the same year, there were 376,000 newly diagnosed cases and 191,000 deaths attributed to colorectal cancer. At present, colorectal cancer has become a public health problem that seriously threatens the lives and health of Chinese residents.

The medical definition of enterostomy is the removal of an already diseased segment of intestine during surgery on a patient, a selected small segment of intestine is pulled out and turned over in the appropriate position in the abdomen, and then sutured onto the abdominal wall to artificially create an open intestinal papilla.^[3,4]^ This procedure is also commonly known as artificial anus. The purpose of enterostomy is to allow intestinal contents to find an exit for excretion, reduce the risk of intestinal obstruction, protect the anastomotic opening of the distal intestine, and promote recovery from intestinal disease.^[4]^ Enterostomy is effective in resolving postoperative defecation problems and prolonging survival.^[5]^ Enterostomy refers to the surgical procedure in which involves securing the opening of the bowel to the abdominal wall by excising the malignant portion of the bowel tube, and performing defecation function instead of the anus. Although the operation saved the patient’s life, it led to changes in the anatomy of the body, the defecation site shifted from the anus to the abdomen, the patient felt fear, loss and even self-loathing, and the patient’s physical function and social interaction were also seriously affected due to the change of defecation mode.^[6]^ For example, physiologically, the patient’s own integrity is destroyed, and postoperative patients may experience loss of defecation function and sexual dysfunction, as well as physical discomfort, including fatigue and insomnia.^[7]^ Psychologically, cancer and postoperative chemotherapy, as stressful events, have become great challenges for patients, and they also need to bear multiple psychological pressures such as body image disorders, self-care ability deficits and social adjustment disorders, and patients are susceptible to experiencing negative emotions such as low self-esteem, loneliness, and depression.^[8]^ In addition, This affects the patient’s social functioning. Due to the existence of stoma, patients need to constantly clean the stoma and clean up stoma excrement every day, which is difficult to play roles in society and family. Coupled with the cost of surgery and long-term purchase of ostomy products, it increases the financial burden of the family, which may lead to patients’ negative thoughts that they are useless, resulting in negative emotions such as low self-esteem and helplessness,^[9]^ and the patient’s self-care ability is reduced. This situation also hampers the postsurgery recovery process and negatively impacts the patients’ overall quality of life.

Targeted psychological interventions can promote the improvement of mental health and reduce negative emotions in patients undergoing enterostomy. Therefore, this study aims to investigate the impact of combining psychological nursing with extended care on alleviating negative emotions and enhancing self-care abilities in patients with colorectal cancer after enterostomy.

## 2. Methods

### 2.1. Research subjects

Approval for this retrospective study was granted by the Ethics Committee. We included 78 patients diagnosed with colorectal cancer who underwent colorectal cancer enterostomy at our hospital between January 2021 and January 2022. Inclusion criteria: Patients who planned to undergo enterostomy; Age ≥18 years; Clear consciousness, able to complete the questionnaire alone or answer the questions correctly; SIS score ≥24 points. Exclusion criteria: Critically ill and unable to cooperate with the research; Impaired cognitive function, affecting communication; Suffering from serious diseases such as the nervous system, immune system, and digestive system, which may affect the results of the research; There is a history of enterostomy in the past; The survival period after discharge is <3 months.

### 2.2. Nursing program

Both groups of patients established files, registering their names, gender, age, home address, occupation, education level, operation time and stoma.

The control group should use routine nursing, which specifically includes routine daily behavior guidance during hospitalization, and complete routine basic nursing operations such as enterostomy nursing according to the doctor’s instructions. Give guidance and suggestions for a series of daily activities such as eating, bathing, exercising, resting, and defecation during treatment. Teaching patients how to clean the enterostomy and surrounding skin, and explaining the possible abnormalities of enterostomy after surgery and emergency measures. After discharge, patients routinely use the opportunity of further consultation for follow-up, and nursing guidance is given to the condition during the revisit.

On the basis of routine nursing, the observation group applied psychological nursing combined with extended care, including: Completing the patient’s psychological intervention in a variety of ways, so that the patient can get reasonable intervention in a personalized way. First of all, informing the patient that there are many people in daily life like them, need to rely on the stoma to maintain life, the stoma itself is not terrible, the person who receives the stoma can participate in social activities normally in addition to having certain differences from normal people physiologically. Patients can continue their usual interests and hobbies in life, and provide patients with real success stories, so that patients can be full of hope and confidence in the future and life. Nursing staff can help patients develop a novel self-awareness through the life meaning therapy, teach patients to have a new understanding and cognition of the value of life through the learning process, and help patients to find the meaning of their lives through the learning process. Nursing staff need to persuade patients on observing more trivia but warm things around them during the intervention process, such as their filial children, beloved grandchildren, the joy of family reunion, the interaction between relatives and friends, etc, so as to keep patients in a happy mood. In the intervention process, patients need to be encouraged to boldly express their inner emotions, and nursing staff need to actively mobilize patients’ emotions, illustrating real cases to patients who received enterostomy, and watch more videos about the friendship association and the relevant lectures for patients had enterostomy. In addition to using the opportunity of further consultation for follow-up, patients are also followed up by other methods, such as telephone follow-up. Tracking the patient’s recovery status, patiently answering and guiding the questions and disposal measures in the rehabilitation, assessing the patient’s psychological state and providing corresponding guidance, and encouraging the patient to face life positively, 1 time/week. Secondly, an extended-care team was established: Patients were instructed to go to the hospital once a month for the follow-up within 6 months after surgery. The further consultation is mainly answered by the nurse in charge and attending doctor that had gave the care to patients in their hospitalization, who guides and nurses the patient’s diet, activities, work and rest patterns and complications, and helps the patient adapt to the life after the stoma. Applying modern communication methods to establish WeChat groups, keeping the doctor-patient contact in a smooth way, and developing the thinking habit which is asking help to the nurses in charge to solve problems spontaneously. Thirdly, about the friendship association: regularly organizing the friendship associations to share patients’ successful self-care experience and realization, and providing timely introduction of new methods and updated products, so that patients can better integrate into society, communicate and support each other, and build confidence in overcoming diseases. Fourthly, the nurse in charge makes follow-up records of the patient with enterostomy that she is responsible for. The content includes the problems encountered in the process of self-care, the mastery of related enterostomy knowledge, and the psychological condition, self-management ability, and the occurrence of related complications of patients. Fifthly, the extended-care team will carry out weekly follow-up record and support the theme meetings, and timely release the correct guidance information or provide the relevant reference materials in WeChat groups for concentrated problems.

### 2.3. Evaluation indicators

1. Anxiety and depression: We compared the changes in anxiety and depression levels before intervention and 3 months postdischarge among patients who underwent enterostomy. Anxiety levels in patients with enterostomy were assessed using the HAMA,^[10]^ while depressed mood was measured using the Hamilton Depression Scale (HAMD).^[11]^
2. Sleep quality: Comparing the changes in sleep quality before intervention and 3 months after discharge. The Pittsburgh Sleep Quality Indicators Scale (PSQI)^[12]^ was employed to evaluate the sleep quality of patients, it comprises a total of 24 questions categorized into 7 components. Each component is scored from 0 to 3 points, with 0 indicating no problem and 3 indicating severe difficulty.
3. Mental toughness: Comparing the changes of mental resilience before intervention and 3 months after discharge. The level of mental resilience in patients had enterostomy was measured by the Resilience Scale in Chinese Edition (CD-RISC),^[13]^ which has a total of 25 items, divided into 3 dimensions, namely the resilience, self-improvement and optimism. The scale comprises a total of 25 items, employing a 5-level scoring system ranging from 0 to 4 points.
4. Self-care ability: The changes in patients’ self-care ability 3 months after discharge were assessed. The Studies of Self-Care Ability Scale (ESCA)^[14]^ was utilized for this purpose. Each question is 0 to 4 points, adding up to a total of 172 points. Higher scores indicate stronger self-care ability.
5. Complications: The incidence of complications within 3 months after discharge was statistically analyzed for both groups, including stoma infection, stoma stenosis, stoma retraction, contact dermatitis, etc.

### 2.4. Statistical analysis

SPSS 22.0 statistical software was utilized for data analysis. Measurement data were assessed for normality and homogeneity of variance. Data, assumed to follow an approximately normal distribution, were presented as mean ± standard deviation and analyzed using the *t* test. Percentage data underwent analysis using Pearson’s chi-square test, Fisher’s exact test, or Mann–Whitney’s test, with statistical significance set at *P* < .05.

## 3. Results

### 3.1. General information

The study included a total of 78 patients diagnosed with colorectal cancer who underwent enterostomy. Among them, the average age of patients was (61.42 ± 9.75) years, 65.38% male and 34.62% female. There were no notable differences observed in the general characteristics between the 2 groups (*P* > .05). For further details, please refer to Table 1.

**Table 1:** Comparison of general information of patients [*x* ± *s*, n (%)].

Item	The observation group (n = 39)	The control group (n = 39)	*t*/X²/*Z*	*P*
Age (years)	60.88 ± 9.93	61.95 ± 9.67	0.481	.632
Gender			0.056	.811
Men	25 (64.10%)	26 (66.67%)		
Women	14 (35.90%)	13 (33.33%)		
Educational background			0.482	.629
Primary school or below	21 (53.84%)	19 (48.72%)		
Secondary school	14 (35.90%)	15 (38.48%)		
University or above	5 (10.26%)	5 (12.82%)		

### 3.2. Comparison of anxiety and depressed mood

**Figure 1.**
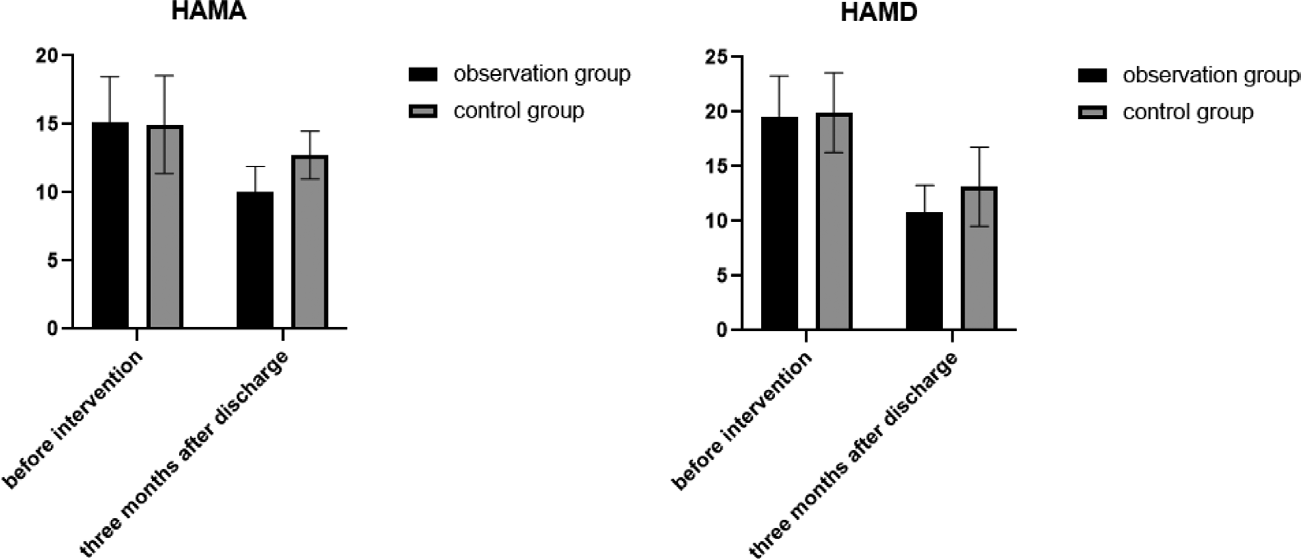
Changes in HAMA and HAMD scores between 2 groups. HAMA = Hamilton Anxiety Scale, HAMD = Hamilton Depression Scale.

Before intervention, HAMA and HAMD scores did not exhibit statistical significance between 2 groups (*P* > .05). Three months after discharge, both groups witnessed a significant decrease in HAMA and HAMD scores compared to their respective scores before the intervention. Moreover, the reduction observed in the observation group was significantly greater than that in the control group (*P* < .05). Kindly refer to Table 2 and Figure 1 for more detailed information.

**Table 2:** Comparison of anxiety and depression mood between 2 groups (*x* ± *s*).

Group	HAMA		HAMD	
	Before intervention	Three months after discharge	Before intervention	Three months after discharge
The observation group (n = 39)	15.12 ± 3.37	10.05 ± 1.86*	19.52 ± 3.79	10.83 ± 2.45*
The control group (n = 39)	14.97 ± 3.58	12.72 ± 1.75*	19.93 ± 3.64	13.16 ± 3.61*
*t*	0.185	6.516	0.491	3.350
*P*	.853	<.001	.625	.001

*Note*: Compared to the situation before intervention.

HAMA = Hamilton Anxiety Scale, HAMD = Hamilton Depression Scale.

* *P* < .05.

### 3.3. Comparison between sleep quality and mental toughness

**Figure 2.**
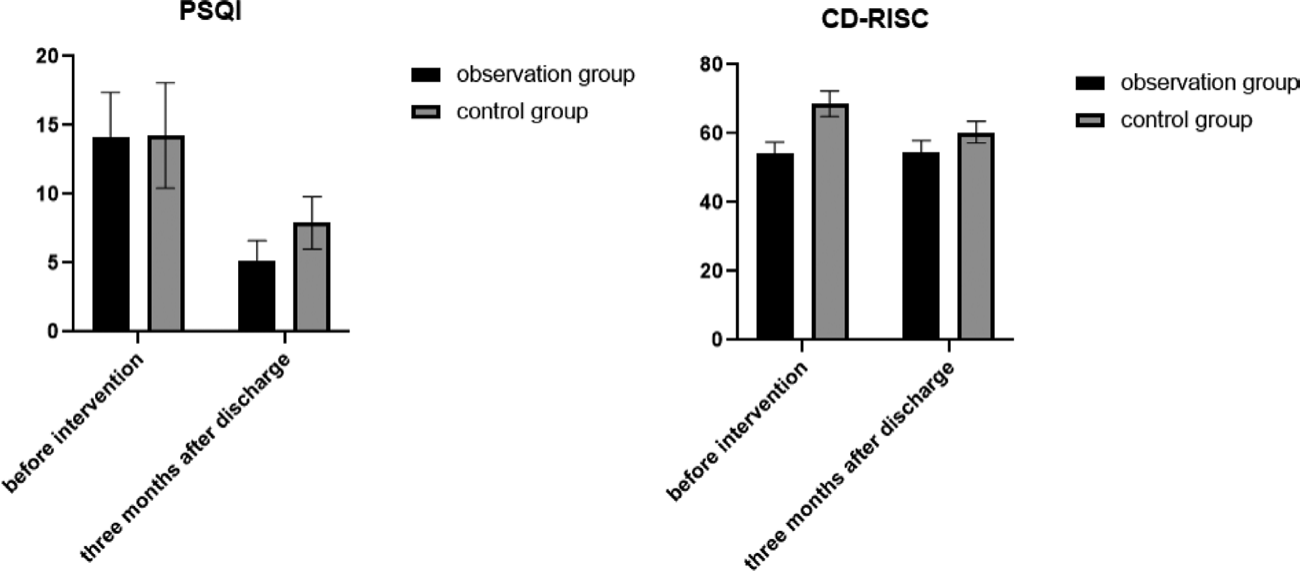
Changes in PSQI scores and CD-RISC scores between 2 groups. CD-RISC = Resilience Scale in Chinese Edition, PSQI = Pittsburgh Sleep Quality Indicators Scale.

Prior to the intervention, the PSQI and CD-RISC scores did not exhibit statistically differences between 2 groups (*P* > .05). Three months postdischarge, both groups exhibited significantly reduced PSQI scores compared to preintervention levels, alongside significantly elevated CD-RISC scores, all showing statistical significance (*P* < .05). Additionally, The PSQI scores were significantly lower and the CD-RISC scores notably higher in the observation group compared to the control group, with statistical significance (*P* < .05). For more detailed information, kindly consult Table 3 and Figure 2.

**Table 3:** Comparison of sleep quality and psychological resilience between these 2 groups (*x* ± *s*).

Group	PSQI		CD-RISC	
	Before intervention	Three months after discharge	Before intervention	Three months after discharge
The observation group (n = 39)	14.16 ± 3.21	5.18 ± 1.44*	54.21 ± 3.23	68.62 ± 3.74*
The control group (n = 39)	14.25 ± 3.82	7.91 ± 1.90*	54.54 ± 3.39	60.37 ± 3.15*
*t*	0.109	7.134	0.435	10.514
*P*	.913	<.001	.664	<.001

*Note*: Compared to the situation before intervention.

CD-RISC = Resilience Scale in Chinese Edition, PSQI = Pittsburgh Sleep Quality Indicators Scale.

* *P* < .05.

### 3.4. Comparison of self-care ability

**Figure 3.**
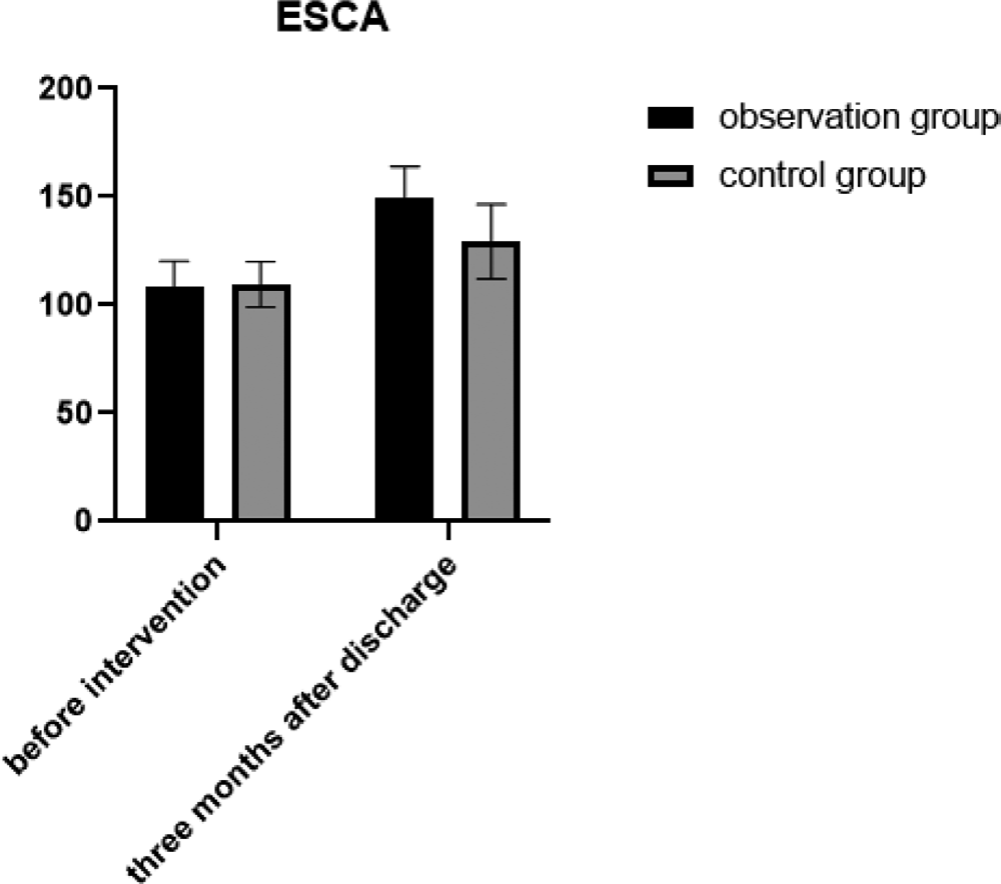
Changes in ESCA scores between 2 groups. ESCA = Studies of Self-Care Ability Scale.

Prior to intervention, no significant difference was observed in ESCA scores between the 2 groups (*P* > .05). Following discharge, both groups showed a significant increase in ESCA scores compared to preintervention levels. Moreover, the observation group displayed a notably higher ESCA score than the control group, with statistical significance (*P* < .05). Refer to Table 4 and Figure 3 for details.

**Table 4:** Comparison of self-care abilities between 2 groups (*x* ± *s*).

Group	n	Before intervention	Three months after discharge	*t*	*P*
The observation group	39	108.74 ± 11.43	149.83 ± 14.08	14.382	<.001
The control group	39	109.61 ± 10.46	129.36 ± 17.21	7.679	<.001
*t*		0.349	5.747		
*P*		0.728	<0.001		

### 3.5. Comparison of the incidence of complications

The overall incidence of complications, including stoma infection, stoma stenosis, stoma retraction, and contact dermatitis in the observation group, was notably lower that in the control group (*P* < .05). Refer to Table 5 for further details.

**Table 5:** Comparison of the incidence of complications between these 2 groups [n (%)].

Group	Stoma infection	Stoma stenosis	Stoma retraction	Contact dermatitis	Total incidence
The observation group (n = 39)	0 (0.00%)	0 (0.00%)	1 (2.56%)	1 (2.56%)	2 (5.12%)
The control group (n = 39)	1 (2.56%)	0 (0.00%)	3 (7.69%)	4 (10.26%)	8 (20.51%)
X²					4.129
*P*					.042

## 4. Discussion

Enterostomy has become a surgical method that currently and frequently been used in clinical practice, and sometimes, it is the only option for treating patients with anorectal diseases. However, as a general patient, it is difficult to accept the fact that he or she will be suffered with a defecation disorder in a short period of time, and the mastery of measures such as the use of artificial stoma and its daily care is not ideal. A considerable proportion of patients have greater psychological stress due to the fact that they cannot accept the artificial stoma,^[15]^ which directly causes the emergence of negative emotions, such as anxiety, nervousness, fear, restlessness, depression, etc, and poor mood state will directly affect the patient’s sleep.^[16]^ Sleep is an important physiological process in human beings, if the quality of sleep cannot be guaranteed, not only the quality of life will be affected, but also the function of multiple systems such as the immune system will be weaken, which in turn will seriously affect the recovery of patients had enterostomy.^[17]^ Changing this situation requires a adapting process of the patient, both physically and psychologically.^[18]^ How to help patients acquiring more relevant knowledge, self-care skills and techniques during hospitalization for enterostomy has become one of the topics in the medical community. Both the active psychological intervention and extended care used in this study have made some efforts in this regard.

Patients had enterostomy need to endure problems such as uncontrollable defecation, intestinal mucosal exposure, leaking feces, and long-term carrying of stomas after surgery, coupled with fear of cancer, treatment costs, changes in the patient’s own image, and living habits caused by emotional and mental pressure, fear of being rejected by relatives or others, showing varying degrees of anxiety and depression.^[19]^ Studies have found that patients with enterostomy have a high proportion of anxiety and depression, which in the long run leads to negative emotions such as fear, depression, and loss of confidence, which if not controlled in a timely and effective manner, such factors can significantly impact both the physical and mental well-being of patients and are detrimental to their postoperative recovery.^[20]^ In this study, 3 months after discharge, the HAMA and HAMD scores in both groups were significantly lower compared to their respective scores before the intervention, and these differences were statistically significant (*P* < .05). This shows that conventional nursing measures and psychological nursing combined extended care can alleviate patients’ anxiety and depression, and the analysis of the reason may be that medical staff can help patients improve anxiety through the guidance of professional knowledge, but depression is an inherent psychological state that requires further psychological intervention to improve. The HAMA and HAMD scores of the 2 groups (*P* < .05), indicating that psychological nursing combined with extended care could better improve anxiety and depression in patients had enterostomy. Enhancing the cognition of the disease in patients had enterostomy, coupled with a certain degree of relaxation training, can help patients to deal with the current anxiety and depression with a more peaceful attitude, so that the body is relaxed, the mind is calmer, and then the positive attitude is increased, and as the patient gradually integrates this method into daily life, the effect is strengthened, and the degree of anxiety and depression is improved.^[21]^

In this study, 3 months after discharge, the CD-RISC scores of the 2 groups were significantly higher than those before the intervention of the same group (*P* < .05), indicating that both routine nursing measures and psychological care combined with extended care could improve the level of mental resilience. The analysis of the reasons may be that the patient is tormented by the disease before surgery and the fear of the unknown operation, coupled with being in the unfamiliar environment of the hospital, which brings a strong impact on the patient’s psychology and cannot adapt well, and with the extension of the postoperative time, with the professional support of medical staff, the patient gradually adapts to the disease and the existence of the stoma, coupled with the support from the family, the patient’s confidence is enhanced and the psychological toughness is improved^[22,23]^; 3 months after discharge. It is explained that through psychological nursing combined with extended care, the training of coping skills for patients can reduce the negative psychological stress response caused by the emergence of unfamiliar environment and stoma, face the disease problem in a positive coping way, enhance their own measures and skills for handling emergencies, effectively control the adverse reactions caused by stressors,^[24–26]^ improve their psychological resilience, and improve their self-care ability.

Generally speaking, from the perspective of positive psychology, mental health can also be called positive mental health, this definition emphasizes that mental health is not only without any problems, but also needs to include the occurrence and enhancement of various positive qualities and positive forces of the individual.^[27,28]^ In the intervention methods of the patients in the control group of this study, on the basis of conventional nursing measures, positive psychological intervention was deliberately emphasized, and positive psychological interventions such as hope therapy, meaning of life therapy, happiness therapy, positive emotional expression and other ways were used to improve the patient’s mood and the patient’s bad emotions, so that they could face the reality of enterostomy in an active, optimistic and positive psychological state. Helping patients correctly understand the lesion, bravely facing themselves, getting out of this negative psychological state of autism and low self-esteem, and reducing the negative impact of stoma on daily life. At the same time, patients can actively recover into society. The improvement of mood can improve the quality of sleep, enhance immunity, and promote the recovery of physical functions. The findings of this study revealed that the sleep quality of both groups showed a certain degree of improvement, However, the observed group exhibited a more significant range of change (*P* < .05), suggesting that the intervention method used by the observation group made the patients more receptive to their self-state, having better psychological adjustment effect, and especially gaining more confidence in their future.

The study still has limitations. The small number of participants selected in this study and all patients from our hospital may have led to some bias in the study results. Secondly, the observation period of this study was only 3 months after the patient was discharged, the limitation of this study was the absence of continuous monitor of the patients’ psychological condition changes and self-care ability after 3 months after discharge. Further expansion of sample size, longer follow-up, improved study design, and further analysis of the results of the study are needed.

In summary, the implementation of psychological nursing combined with extended care demonstrates significant potential in alleviating negative emotion among patients with colorectal cancer after enterostomy. Furthermore, it enhances sleep quality, psychological resilience, and self-care ability while reducing the incidence of complications. These findings hold considerable value for promotion and application in clinical settings.

## Author contributions

**Conceptualization:** Kun Yao.

**Data curation:** Kun Yao.

**Formal analysis:** Kun Yao.

**Investigation:** Kun Yao.

**Methodology:** Kun Yao.

**Writing – original draft:** Kun Yao.

**Writing – review & editing:** Kun Yao.
